# A homozygous *PIWIL1* frameshift variant triggers azoospermia and reveals a strong selective constraint on germline genome integrity

**DOI:** 10.1007/s10815-026-03826-0

**Published:** 2026-02-18

**Authors:** Aissatu Balde-Camara, Morgane Le Beulze, Anna Lokchine, Laura Mary, Céline Cierniewski, Linda Akloul, Lucas Freton, Céline Pimentel, Anne-Laure Barbotin, Gabriel Livera, Nathalie Rioux-Leclercq, Sylvie Jaillard, François Vialard

**Affiliations:** 1 RHuMA, UFR-SVS, UVSQ, Montigny le Bretonneux, France; 2 Université Paris-Saclay, UVSQ, Inserm, IMPROVE, Versailles, France; 3 Laboratory of Development of the Gonads, Université Paris-Saclay, CEA, DRCM/IBFJ, Fontenay-aux-Roses, France; 4 Laboratoire de Cytogénétique et Biologie Cellulaire, CHRU Rennes, Rennes, France; 5 Irset, UMR S 1085, Univ Rennes, Rennes, France; 6 Laboratoire d’Anatomopathologie, CHI de Poissy St Germain en Laye, Poissy, France; 7 Service d’Urologie, Hopital de la Sagesse, Rennes, France; 8 Centre de Fertilité d’Armor et HPCA, Plérin, France; 9 Institut de Biologie de La Reproduction-Spermiologie-CECOS, Hôpital Jeanne de Flandre, CHU Lille, Université de Lille, Lille, France; 10 Genetic Stability Stem Cells and Radiation, Université Paris Cité, CEA, Fontenay-aux-Roses, France; 11 Laboratoire d’Anatomopathologie, CHU de Rennes, Rennes, France

**Keywords:** Azoospermia, PIWIL1, PiRNA, Genome integrity, Maturation arrest, Miwi, Transposon, Selection pressure

## Abstract

**Introduction:**

PIWI proteins and their associated piRNAs constitute a highly conserved pathway that is essential for maintaining germline genome integrity through transposon silencing, viral defence, and RNA protection in germ cells. In mammals, defects in this pathway might lead to meiotic failure and infertility. However, the contributions of individual PIWI genes to human spermatogenesis remain poorly defined.

**Case report:**

Here, we report on a 29-year-old man with non-obstructive azoospermia and found to carry a homozygous frameshift mutation in *PIWIL1* (NM_004764.5:c.1176_1179delAACT). Histological examination of testicular tissue revealed spermatogenic arrest at the spermatocyte stage, and immunohistochemistry confirmed the absence of the full-length PIWIL1 protein and the absence of spermatids. Considering the low reported variant frequency for such a highly conserved mechanism, we evaluated the genetic constraints on *PIWIL1.*  A comparison of mRNA and amino acid sequences in the human vs. the great apes revealed the likely presence of purifying selective pressure.

**Discussion:**

Taken as a whole, these data reinforce the body of evidence showing that PIWIL1 is essential for human spermatogenesis and highlight the importance of the piRNA pathway in safeguarding the male germline. Defects in genome defence mechanisms might therefore constitute an underexplored cause of human male infertility.

## Introduction

The process of gametogenesis faces various challenges. The best known steps are germline stem cell maintenance, the halving of the number of chromosomes during meiosis, and the spermiogenesis leading to spermatozoa. Less well known steps occur during gametogenesis, including (i) protection of mRNAs (to ensure ongoing transcription and translation processes after genome inactivation during and after the histone-protamine transition that completes spermiogenesis), (ii) transposon silencing (to ensure genome integrity), and (iii) viral defence (to avoid genome integration). All these processes appears to be wholly or partly linked to the P-element-induced wimpy (PIWI)/PIWI-interacting RNAs (piRNAs) [1, 2] for protection of genome integrity and for protein regulation in germ cells [3]. PIWI proteins and piRNAs are commonly perceived to be germline-specific, even though a somatic function has been documented [4]. Furthermore, piRNAs and PIWI proteins are highly conserved and found in the majority of animals [5], including vertebrates, arthropods, and nematodes (see Ensembl: https://www.ensembl.org/index.html).

PIWI proteins are essential for gametogenesis in animals [6, 7]. In *Drosophila*, the three Piwi proteins Argonaute, Aubergine, and Piwi are required for germ cell formation and germline stem cell maintenance in both males and females [5, 6]. piRNAs were first identified in the fly testis as a novel class of “small-interfering RNAs” [8] that guided PIWI proteins to cleave target RNA, promote heterochromatin assembly, and methylate DNA.

The mouse genome encodes three *Piwi* paralogs (*Miwi*, *Mili*, and *Miwi2*), whereas the human genome encodes four *PIWI* paralogs: *PIWIL1* (*HIWI*), *PIWIL2* (*HILI*), *PIWIL3* (*HIWI3*), and *PIWIL4* (*HIWI2*) [9]; all are highly expressed in the testis. Piwi proteins comprise four distinct domains: the PAZ and MID domains for piRNA loading, the PIWI C-terminal domain with RNase H activity [10–12], and the N-terminal domain (N-domain) that binds to Tudor-domain-containing proteins (TDRDs). TDRDs act as scaffolds to form the core of the chromatoid body, a cytoplasmic, non–membrane-bound organelle found in haploid, round spermatids [13, 14]. The N-domain also contains a conserved destruction box (D-box), whose signature is shared by substrates of APC/C ubiquitin E3 ligase.

The most abundant piRNA population in mammals corresponds to pachytene piRNAs, with more than 5 million molecules per spermatocyte [15]. Although pachytene piRNAs cleave hundreds of RNAs, a change in steady-state level is only detectable for a small fraction of transcripts. The cleavage of the few targets whose abundance is reduced significantly by piRNAs might be essential for male fertility – even though the targets’ exact functions are still unknown; it has been suggested that piRNAs are involved in gene regulation, but this is subject to debate [16]. The emergence of these PIWI/piRNA complexes reflects an evolutionary conflict between transposons (which must integrate into germline DNA to ensure their propagation) and the host genome (which, in order to maintain germline integrity, must protect itself from transposon-encoded proteins). During evolution, transposons have participated in genome expansion and have contributed to biological diversity. Transposon insertion can lead to chromosome rearrangement by homologous recombination and/or modifying gene expression in time or space. To reset the epigenome and erase genomic imprinting, gametogenesis requires germline reprogramming [17]. Erasing DNA methylation causes a burst of transposon transcription, which the PIWI/piRNA complex must oppose. It is noteworthy that PIWI/piRNAs reportedly regulate protein-coding genes in mouse spermatids.

In various species, *PIWI* genes have essential roles in the germline, and knock-out leads to distinct fertility phenotypes. In the golden hamster, both females and males lacking *Piwil1* are sterile [18]; the zygotes are arrested at the two-cell stage in females, and males display spermatocyte arrest. *Piwil2* and *Piwil4* knock-out only result in male infertility, through spermatocyte arrest and spermatid arrest, respectively. In contrast, female *Piwil3*-knock-out hamsters displayed reduced fertility with significantly fewer offspring and the delayed development of embryos [19].

In mice, *Miwi* (*Piwil1*), *Mili* (*Piwil2*), and *Miwi2* (*Piwil4*) are all essential for male fertility, but female knock-out mice are unaffected; hence, PIWI function is sex-specific in this species. *Miwi*-null mutant males display spermatogenesis arrest at the early round spermatid stage after meiosis [20], while *Mili* and *Miwi2* are arrested at the pachytene stage during meiosis [21, 22].

In other species, *PIWI* genes also appear to have a major or prominent role; for example, null male chickens [23] and zebrafish [24] are often sterile (for a review see [25]).

In humans, *PIWIL1* heterozygous missense variants have been identified in the Genome Aggregation Database (gnomAD, https://gnomad.broadinstitute.org). In a mouse model mimicking a complex human variant identified in a small Chinese cohort of infertile men, males were reportedly sterile and had few condensed spermatids [26]; this situation is quite similar to that observed with *Miwi* knock-out mice. However, this work is subject to debate and has not been replicated. Furthermore, no *de novo* mutations were observed in a much larger international cohort [27]. Furthermore, the *PIWIL1* variant c.1580G > A, p.Arg527Lys (rs1106042) is reportedly associated with non-obstructive azoospermia (NOA; odds ratio [95% confidence interval] = 4.737 [1.314–17.072]) [28]. In men, low *PIWIL2* mRNA expression is associated with a low sperm count, and elevated *PIWIL1* mRNA expression is associated with low progressive motility of ejaculated spermatozoa [29].

Recently, a homozygous stop-gain mutation in *PIWIL1* (c.688C > T, pArg230*) was described in a man presenting a round spermatid arrest, and two homozygous missense variants in *PIWIL2* were described for patients with Sertoli-cell-only syndrome (c.839A > C; p.Tyr280Ser) and azoospermia lacking a documented histological phenotype (c.1697G > A, p.Arg566His) [30].

Here, we report on the observation and *in silico* validation of a new homozygous frameshift mutation in *PIWIL1* (c.1176_1179delAACT; p.Thr393fs). Furthermore, given the importance of the mechanism driven by the PIWI gene family in spermatogenesis and the gene’s high level of conservation among the mammals, we compare the mRNA and protein sequences in the human vs. the great apes.

## Case report

### Clinical presentation

A 29-year-old man consulted at Pontchaillou Hospital (part of Rennes University Medical Centre, Rennes, France) for NOA. The azoospermia was confirmed twice, and the ejaculate volume was normal. The patient’s medical history was unremarkable. Serum hormone assays gave values of 15.2 IU/L for FSH (normative range: 1.55–9.74), 26.0 g/mL for inhibin B (normative range: 80–325), and 7.25 nmol/L for total testosterone (normative range: 1.32–8.13). Genetic testing showed a normal karyotype and the absence of Y chromosome microdeletions.

An ultrasound examination of the scrotum indicated that the patient’s testicles were intrascrotal. Bilateral hypotrophy was noted, with volumes of 6.2 and 6.0 ml. The two testicles displayed uniform echogenicity, with no focal lesions, colour Doppler signal asymmetry, or microcalcifications. The patient’s epididymes were symmetrical in shape and of similar size, with clear visualization of the head, body, and tail of the epididymis, the flexure of the tail of the epididymis, and the vas deferens on both sides. Importantly, no signs of varicocele were observed – even during the Valsalva manoeuvre. The cord structures’ morphological features were intact. Furthermore, the right and left seminal vesicles and the ampullae of the ductus deferens were symmetrical.

### Genomic analysis and patient follow-up

The patient gave his written, informed consent to genetic testing, which included whole-genome sequencing. Strict filters were applied, in order to select only rare variants in genes encoding proteins that were involved in spermatogenesis or enriched in the testes, according to the Gene-Tissue Expression (https://gtexportal.org/home/), Human Protein Atlas (https://www.proteinatlas.org), PubMed (https://pubmed.ncbi.nlm.nih.gov/), and Ensembl databases.

We identified a homozygous frameshift variant in *PIWIL1*: c.1176_1179delAACT; p.Thr393Ilefs*9 (NM_004764.5), which was absent from gnomAD. Considering that (i) the *PIWIL1* gene is highly conserved in vertebrates, (ii) *Miwi* knock-out in the mouse is associated with spermatogenic arrest at the beginning of the round spermatid stage and leads to male infertility, and (iii) a pathologic frameshift variant has been reported previously [30], we considered that this variant was probably pathogenic. No other variants of interest were identified.

After genetic counselling and even though the predicted likelihood of sperm retrieval was low, the patient decided to undergo testicular sperm extraction. No spermatozoa were retrieved, and the results of the histological analysis argued in favour of spermatogenesis arrest at the spermatocyte stage.

### *In silico* modelling of the p.Thr393Ilefs*9 PWILI1 protein

**Fig. 1:**
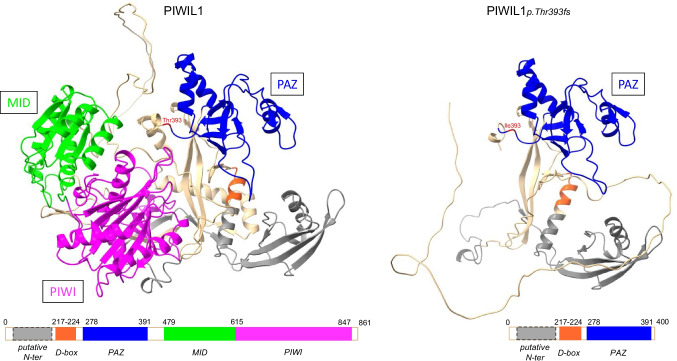
*In silico* modelling of human PIWIL1- WT and PIWIL1-p.Thr393Ilefs*9. Modelling structure of the human PIWIL1 with the four main domains: N-terminal, PAX, MID, and PIWI. The WT and mutated amino acids are represented in red

Even though the variant is located in exon 11 and is predicted to cause nonsense-mediated decay of the mRNA (leading to the absence of protein), we generated a theoretical representation of the protein structure in the unlikely case in which the mutant transcript escapes nonsense-mediated decay and produces a truncated protein*.* The structure of human PIWIL1 was predicted with AlphaFold2 (https://alphafold.ebi.ac.uk/) [31] and ChimeraX 1.9 (https://www.rbvi.ucsf.edu/chimerax/) software (Fig. 1). The wild type and mutant PWILI1 proteins were compared with regard to the four main domains: MID, PIWI, PAZ, and the N-domain (containing the D-box). The frameshift variant led to a deletion just after the PAZ domain (amino acids 278 to 391, according to the UniProt database (https://www.uniprot.org/uniprotkb/Q96J94/entry#family_and_domains) and thus the complete absence of the MID domain (amino acids 479 to 615) and the PIWI domain (amino acids 555 to 847). The loss of these two domains in p.Thr393Ilefs*9 PWILI1 might modify or abolish the protein’s functions and recruitment or might induce early degradation.

### The *PIWIL1-*c.1176_1179delAACT variant disrupts meiotic progression and germ cell differentiation in human testes

**Fig. 2:**
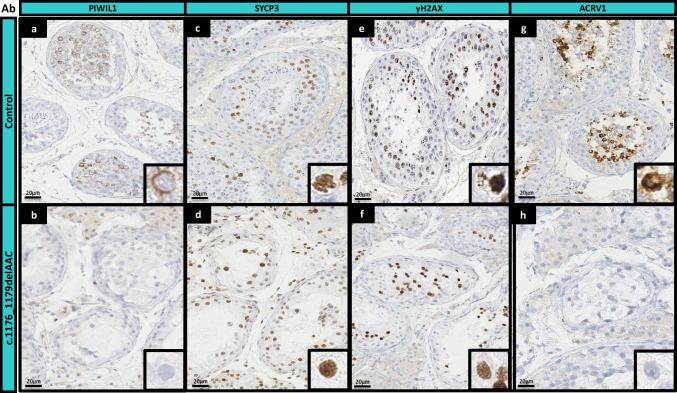
Immunohistochemical staining of seminiferous tubules. PIWIL1 expression in (**a**) control and (**b**) patient with NOA and a PIWIL1 mutation. SYCP3 expression in (**c**) control and (**d**) patient with NOA and a PIWIL1 mutation. γΗ2AFX expression in (**e**) control and (**f**) patient with NOA and a PIWIL1 mutation. ACRV1 expression in (**g**) control and (**h**) patient with NOA and a PIWIL1 mutation. Scale bars = 20 μm

In light of the previous case report on the homozygous pArg230* variant and the significantly lower amounts of piRNAs (suggesting that the variant is pathogenic), we investigated the impact of the *PIWIL1*-c.1176_1179delAACT variant on human spermatogenesis and performed immunohistochemical analyses of the patient’s testicular tissue. The patient’s results were compared with those of a control individual with normal spermatogenesis. Firstly, the presence of PIWIL1 was probed with an antibody specific for amino acids 405 to 493 (reference: HPA066880, Merck, Waltham, MA, USA). In the control, PIWIL1 was strongly expressed in spermatocytes and was predominantly located in the cytoplasm; this was consistent with the protein’s role in the piRNA-mediated regulation of transposons during meiosis (Fig. 2a). In contrast, PIWIL1 expression was not observed in the patient with the *PIWIL1*-c.1176_1179delAACT mutation (Fig. 2b). Given that the HPA066880 antibody does not bind to the truncated protein, this result confirmed the absence of the MID and PIWI domains and is consistent with the complete loss of protein function (due to RNA decay or to expression of the truncated protein).

To characterize the progression of spermatogenesis through defined stages, a three-antibody panel was applied (Table 1). No difference between the patient and the control was observed in the expression of synaptonemal complex protein 3 (expressed in spermatocyte I stage; Fig. 2c and d). However, the expression of γH2AX (a marker of the early spermatocyte stage; Fig. 2e and f) was much lower in the case than in the control. The absence of spermatids was confirmed. ACRV1 (which localizes to the acrosome of round spermatids; Fig. 2g and h) could not be detected in the patient’s tissue.

**Table 1:** Primary antibodies and experimental conditions used in immunohistochemistry experiments

Target	Species	Reference	Supplier	Human protein atlas approved	Dilution
PIWIL1	Rabbit	HPA066880	Sigma-Merck	Yes	
SYCP3	Rabbit	HPA043938	Sigma-Merck	Yes	800e
γH2AX	Rabbit	HPA051647	Novus	Yes	50e
ACRV1	Rabbit	HPA038718	Sigma-Merck	Yes	800e

Taken as a whole (and even though functional studies have not been performed), these results suggest that the *PIWIL1* c.1176_1179delAACT variant may cause meiotic arrest and thus the absence of post-meiotic germ cells. These findings in human tissue are consistent with the data from murine models (in which *Miwi* knock-out leads to meiotic arrest) and underscore the conserved role of PIWI proteins in germ cell development from one species to another.

### Evolutionary constraints on PIWIL1

We then evaluated the evolutionary constraints on PIWIL1, considering that (i) the human phenotype is quite similar to that in other species and (ii) there are few patients with homozygous frameshift or stop-gain variants. Firstly, we compared the mRNA and protein sequences in the human vs. the great apes (Table 2). For proteins, the percentage homology ranged from 99.07% for the Sumatran orangutan (eight amino-acid difference) to 99.77% for gorilla (one amino-acid difference). Using the method described by Nei & Gojobori [32], we calculated the number of nonsynonymous substitutions per nonsynonymous site for the human sequence vs. the great ape sequences (dN); this indicates the changes that affect protein sequences. Secondly, we calculated the number of synonymous substitutions per synonymous (dS) site for two sequences, which serve as a neutral reference for evolutionary processes. A dN/dS ratio below 1 suggests purifying selection, in which protein-altering mutations are selected against. A dN/dS ratio above 1 suggests positive selection, in which protein-altering mutations are favoured. In all cases, the ratio was below 1 and ranged from 0.05 (for the gorilla) to 0.23 (for the chimpanzee).

**Table 2:** Great Apes’ sequence homology compared to human and evolutionary constraint evaluation

Species		Bonobo (*Pan paniscus*)	Chimpanzee (*Pan troglodytes*)	Gorilla (*Gorilla gorilla*)	Sumatran orangutan (*Pongo abelii*)
% homology with human sequence		99.54%	99.54%	99.77%	99.07%
Number of amino-acids change		4	4	1	8
Number of mRNA nucleotides changes	Synonymous	4	5	11	37
	Non synonymous	5	4	1	8
dN (nonsynonymous substitution rate)		0.0060	0.0060	0.0030	0.0120
dS (synonymous substitution rate)		0.0321	0.0266	0.0599	0.2171
dN/dS		0.19	0.23	0.05	0.06

## Discussion

This is the second ever report on a patient with a maturation arrest at the spermatocyte pachytene stage and a homozygous frameshift variant in *PIWIL1*. This homozygous variant is predicted to be deleterious because the frameshift is frequently associated with RNA decay. Even though the production of a truncated protein was unlikely in our patient, the immunostaining results and *in silico* modelling indicated that a putative truncated protein would lack the MID and PIWI domains at least (Figs. 1 and 2). The MID domain is required for binding to the uridine in the first position of piRNAs, and the PIWI domain performs the double-stranded-RNA-guided hydrolysis of single stranded-RNA, as has been observed for the Argonaute family of related proteins [33]. Considering that the antibody probe used here is specific for amino acids 405 to 493 and that we cannot be certain about RNA decay, the truncated protein (if it exists) retains the N-domain (that binds to TDRDs, contains a D-box and acts as a recognition signal for binding to the APC/C complex) and the PAZ domain (which specifically binds the 2′-O-methylated 3′-end of piRNAs). We would not expect the truncated protein to be functional.

Lastly, one can hypothesize that *PIWIL1* acts as a recessive, pathogenic gene in male infertility. This hypothesis is strengthened by the observation that males who are heterozygous for missense variants and even frameshift variants are fertile and that no homozygous *PIWIL1* loss-of-function (LoF) variants have been reported in gnomAD. The most frequent heterozygous LoF variant (reported 50 times) is p.Arg230*, which is associated with round spermatid arrest when homozygous [30]. One can reasonably hypothesize that whatever the size of the truncated protein, a LoF mutation is highly damaging for germline genome integrity. Furthermore, the probability of being LoF-intolerant is null, which confirms the recessive model of transmission.

With a view to evaluating a putative genetic constraint on *PIWIL1*, we compared the human and the great apes with regard to the mRNA and protein sequences. As expected, there were few differences; this strengthens the hypothesis whereby *PIWIL1* is subject to negative, purifying, selective pressure. Further research is needed to localize variants and evaluate their impact on protein function.

As suggested previously [27], *PIWIL1* does not appear to be frequently mutated in human male infertility or in human female infertility. Furthermore, there are fewer LoF variants than expected in gnomAD: 55 have been identified, whereas 87.4 are predicted. We hypothesize that the *PIWIL1* gene is subject to negative pressure and that the protein structural variant has a negative impact. A transcript that lacks its expected variation is supposedly constrained. To assess constraints, three scores have been developed in gnomAD: the loss-of-function observed/expected upper bound fraction (LOEUF) and the Z-scores for synonymous and missense variants (for more details of these scores, see the gnomAD website: https://gnomad.broadinstitute.org/help/constraint). Although the gnomAD Z score for synonymous variants is as expected for *PIWIL1* (Z = 0.39; 333.1 expected variants vs 320 observed variants), the situation is different for the missense variants: the Z-score is 4.17, with 956.8 expected variants vs 604 observed variants. This suggests that the heterozygous variant might have a negative impact on spermatogenesis (and probably also on oogenesis) and might predispose to infertility.

The above findings contrast with Gou et al.’s report on D-box variants in PIWI proteins, in which mutations identified as *de novo* in an infertile man were modelled in the mouse. The researchers hypothesized that the genetic defects were directly responsible for male infertility [26]. Together with the data in gnomAD, our present data indicate that a heterozygous variant may predispose to (but not cause) infertility. This is in line with the results observed for the *PIWIL1* variant c.1580G > A, p.Arg527Lys (rs1106042) associated with NOA (odds ratio [95% confidence interval] = 4.737 [1.314–17.072]) [28].

Although many disease-causing variants are associated with defects in chromosome breakage and repair (e.g. in *MEIOB*, *MEI1*, and *SPATA22*) or synapsis formation (e.g. in *STAG3*), loss of germline-protecting genome integrity is also a cause of male infertility. Until recently, only pathogenic variants in *PIWIL1* partners had been reported in literature. However, this might have been due to the need for strong conservation of the protein sequence; variations would be associated with less efficient spermatogenesis but probably also less efficient gametogenesis. This phenomenon might concern other genes associated with infertility in humans or other mammals. Further studies will be needed to answer this question and to evaluate the potential negative impact of missense or LoF variants on gametogenesis and their putative role in predisposing to infertility.

Taken as a whole, these data reinforce the body of evidence showing that PIWIL1 is essential for human spermatogenesis. Furthermore, the data highlight the importance of the piRNA pathway in safeguarding the male germline. Defects in genome defence mechanisms might therefore constitute an underexplored cause of human male infertility.

## Data Availability

No datasets were generated or analysed during the current study.
